# Evaluation of the Efficacy of Remote Ischemic Preconditioning in Reducing Renal Injury in Patients Undergoing Partial Nephrectomy: A Systematic Review

**DOI:** 10.7759/cureus.92385

**Published:** 2025-09-15

**Authors:** Abhinav Singhal, Maanya Bhardwaj, Gaurika Bhardwaj, Sachin Yallappa

**Affiliations:** 1 Urology, University Hospitals Birmingham NHS Foundation Trust, Birmingham, GBR; 2 Urology, Cambridge University Hospital NHS Foundation Trust, Cambridge, GBR; 3 Critical Care, Royal Marsden, London, GBR

**Keywords:** egfr, ischemia-reperfusion injury, kim-1, ngal, partial nephrectomy, remote ischemic preconditioning, renal function, urinary biomarkers

## Abstract

Partial nephrectomy often results in temporary renal ischemia, predisposing the kidney to ischemia-reperfusion injury. Remote ischemic preconditioning is a novel technique that has emerged as a potential strategy to attenuate renal ischemia-reperfusion injury. Remote ischemic preconditioning involves brief, controlled ischemia of a limb, which induces the development of systemic protective mechanisms against ischemia. This systematic review evaluates the efficacy of remote ischemic preconditioning in reducing renal injury post-partial nephrectomy, focusing on urinary biomarkers, renal function parameters and long-term kidney function outcomes.

This review followed Preferred Reporting Items for Systematic Reviews and Meta-Analyses (PRISMA) guidelines and comprehensive literature searches were performed across PubMed, EMBASE, Cochrane and SCOPUS for studies published from 1st January 2015 to 15th June 2025. Eligible studies included randomized controlled trials, cohort and case-control studies examining remote ischemic preconditioning in adult patients undergoing partial nephrectomy, with outcomes involving urinary biomarkers such as neutrophil gelatinase-associated lipocalin (NGAL) and renal function parameters such as estimated glomerular filtration rate (eGFR) and serum creatinine. Risk of bias was assessed using frameworks such as Cochrane’s RoB 2.0, ROBINS-I, and Newcastle-Ottawa Scale.

The results were mixed with five studies meeting the inclusion criteria, comprising four randomised controlled trials and one cohort study. High-quality trials demonstrated significant short-term improvements in postoperative eGFR, reduced pain and shorter hospital stays when remote ischemic preconditioning was used as part of a multimodal strategy. Others showed reduced urinary NGAL or attenuated serum creatinine rise, but without consistent functional benefit. Variability in remote ischemic preconditioning protocols, outcome measures, and patient populations limited direct comparisons. Overall, studies were at low to moderate risk of bias.

Remote ischemic preconditioning appears to be a safe and feasible intervention with potential short-term renal benefits following partial nephrectomy. However, evidence remains inconclusive due to heterogeneity and limited long-term data. Future large-scale, standardized trials incorporating sensitive biomarkers and robust renal function outcomes are needed to clarify the clinical utility of performing remote ischemic preconditioning and optimizing its application in renal surgery.

## Introduction and background

Partial nephrectomy is increasingly performed for localized renal tumors to preserve renal function while maintaining oncologic control [1]. However, the procedure often requires temporary clamping of the renal vessels, leading to ischemia-reperfusion injury (IRI) [1]. IRI is a paradoxical phenomenon in which the restoration of blood flow to previously ischemic tissue triggers a cascade of metabolic disturbances that cause cellular injury and death [2]. This process initiates a complex inflammatory response characterized by the generation of reactive oxygen species and neutrophil adhesion to the endothelium with subsequent release of pro-inflammatory cytokines [2]. Biomarkers such as neutrophil gelatinase-associated lipocalin (NGAL) and functional indices like estimated glomerular filtration rate (eGFR) are frequently used to measure the extent of renal injury [3]. Postoperative acute kidney injury (AKI) occurs in up to 20-30% of patients following partial nephrectomy, and a subset of these patients progress to chronic kidney disease (CKD), which is associated with higher cardiovascular risk and reduced long-term survival [4,5]. These epidemiologic data highlight the need for strategies to mitigate IRI during renal surgery. 

Remote ischemic preconditioning (RIPC) is a non-invasive technique first described in cardiology, where brief cycles of limb ischemia using a blood pressure cuff were shown to protect the heart from ischemic injury during surgery [6]. Typically, three to four cycles of five minutes of cuff inflation followed by reperfusion are applied to a limb, triggering systemic protective pathways via neural and humoral mechanisms [6,7]. Since its discovery, RIPC protocols have been tested across multiple surgical disciplines, including cardiac, vascular, and abdominal surgery [6,7]. In renal surgery, the hypothesis is that RIPC may reduce ischemia-reperfusion-related tubular injury during partial nephrectomy, thereby preserving renal function. 

Preventing reperfusion injury is of clinical significance in surgical fields such as organ transplantation and tissue transfer, where minimizing tissue damage can influence procedural success and long-term outcomes [8]. This novel technique has emerged as a promising, non-invasive intervention. Several clinical trials have investigated the role of RIPC in renal surgery, but results have been inconsistent, and its effect on renal biomarkers and long-term kidney function remains unclear. This systematic review aims to evaluate whether RIPC reduces renal injury in patients undergoing partial nephrectomy, as assessed by urinary biomarkers and long-term renal function outcomes.

## Review

Methods

This systematic review was conducted in accordance with the Preferred Reporting Items for Systematic Reviews and Meta-Analyses (PRISMA) guidelines [9]. Studies were selected according to the inclusion and exclusion criteria provided in Table 1. 

**Table 1:** Exclusion and inclusion criteria for the studies eGFR: estimated Glomerular filtration rate, NGAL: neutrophil gelatinase-associated lipocalin

	Inclusion	Exclusion
Population	Paper only includes adults >18 years of age, Patients undergoing partial nephrectomy	Any other surgery, Paediatric patients <18 years
Study design	Randomised controlled trials, Prospective or retrospective cohort studies, Case control studies, Case reports >20 patients	Case reports <20 patients, Abstracts, Systematic reviews, Metanalysis, Editorials
Language	Papers in English	Papers not in English
Time frame	Articles published between 1st January 2015 – 15th June 2025	Articles outside this time frame
Outcomes	length of hospital stay, readmissions, urinary biomarkers (NGAL) and renal function parameters (eGFR & serum creatinine)	No relevant clinical data

A comprehensive search was conducted in the following electronic databases: PubMed, EMBASE, Cochrane Central Register of Controlled Trials (CENTRAL) and SCOPUS. Articles published from 1st January 2015 to 15th June 2025 were included in the search. A structured search strategy was developed using keywords and Medical Subject Headings (MeSH) terms. The search terms included combinations of partial nephrectomy, nephron sparing surgery, remote ischemic preconditioning, remote ischemic conditioning, renal injury, urinary biomarkers, NGAL, serum creatinine or kidney function. Full search strategies used for the databases are available in Appendix A. The search strategy was limited to bibliographic databases. Grey literature sources and clinical trial registries, for instance ClinicalTrials.gov and conference proceedings, were outside the scope of this review and therefore not systematically searched, which may have led to omission of unpublished or ongoing studies. 

**Figure 1:**
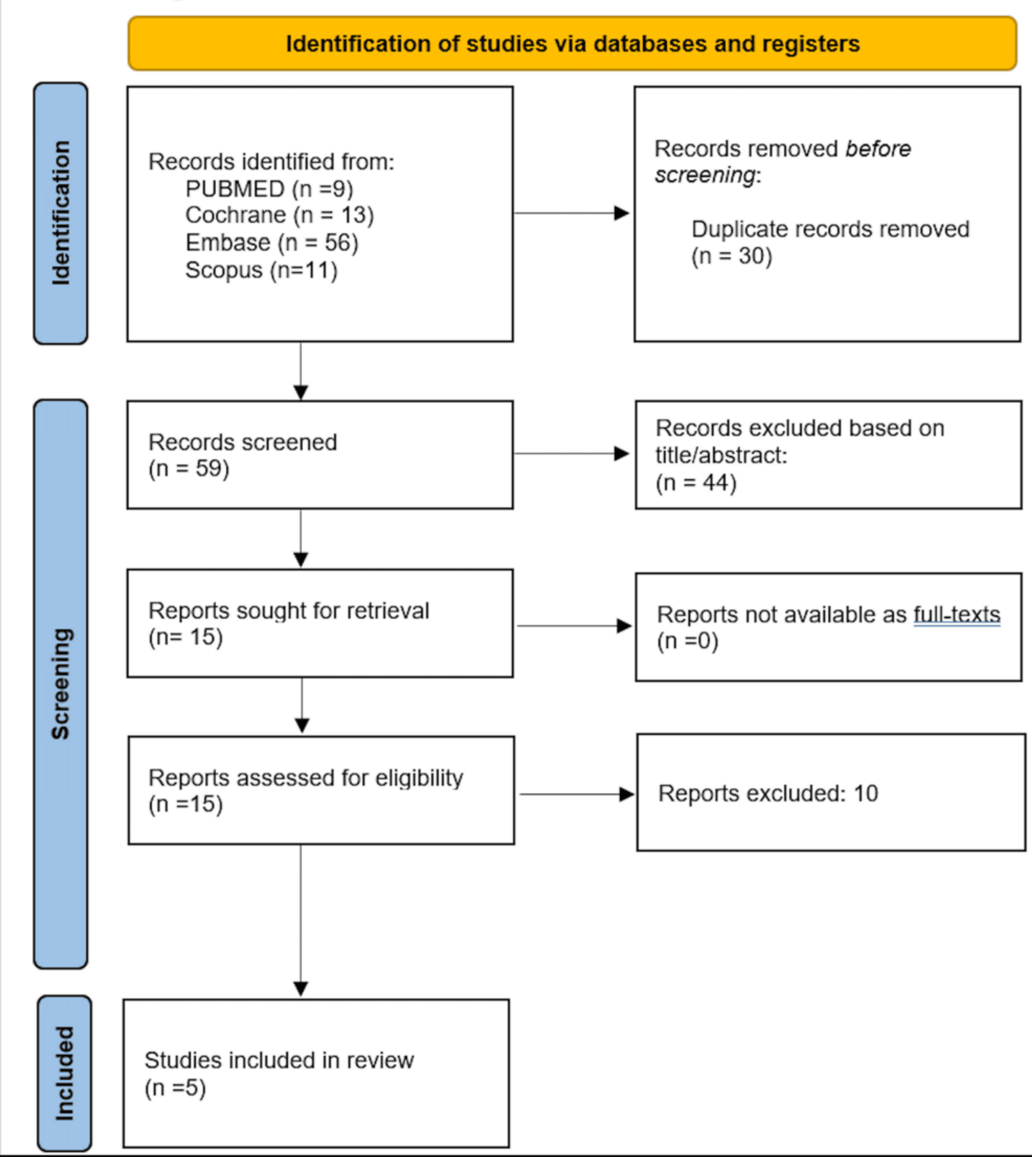
Preferred Reporting Items for Systematic Reviews and Meta-Analyses (PRISMA) flow diagram for identifying studies for inclusion in systematic review

All search results were imported into the Rayyan (Qatar Computing Research Institute (QCRI), Doha, Qatar) web application for systematic review management, and duplicate records were automatically identified and removed using its de-duplication function, followed by manual verification. Titles and abstracts were screened independently by three reviewers using the defined inclusion and exclusion criteria. Full texts for potentially eligible studies were retrieved and assessed for final inclusion. The PRISMA flow diagram in Figure 1 provides an overview of the process used for selecting studies for the review [9]. 

Data was extracted independently by three reviewers using a standardized form. The following data was collected including study characteristics, population details, intervention details, comparator details, outcome measures, key findings and conclusions. The methodological quality of included studies was assessed using Cochrane Risk of Bias 2.0 tool [10] for randomized controlled trials, ROBINS-I [11] for non-randomized studies, and the Newcastle-Ottawa scale [12] for cohort and case control studies. Each domain was rated as low, moderate, or high risk of bias. Discrepancies were resolved by consensus and discussion amongst the three reviewers. 

Although heterogeneity in protocols and outcomes was expected, we first attempted an exploratory meta-analysis of comparable continuous outcomes reported by randomized trials. We extracted group means, standard deviations (or converted SEs/CIs to SDs where required) and sample sizes. Mean differences (intervention − control) were pooled using a random-effects model (DerSimonian-Laird). Heterogeneity was quantified with Cochran’s Q, I², and τ². We pre-specified that an I² > 75% would be considered high heterogeneity and would prompt abandonment of pooling for that outcome; moderate heterogeneity (I² 30-75%) would be reported but interpreted cautiously. Where outcomes were reported at different timepoints (e.g., earliest 48 hours versus three months), we considered pooling exploratory and reported it solely to show the magnitude and precision of pooled estimates; findings were triangulated with a Synthesis Without Meta-analysis (SWiM)-compliant narrative synthesis. Effect-direction coding in the narrative synthesis defined higher eGFR (or smaller creatinine rise/lower NGAL) as favourable. When results conflicted, we prioritised evidence from trials with lower risk of bias and larger sample sizes. 

Due to heterogeneity in study designs and outcome measures, a narrative synthesis was conducted following the SWiM reporting guideline, with studies grouped thematically by intervention characteristics and outcome type [13]. Reference management was conducted using EndNote. Data extraction and tabulation were performed in Microsoft Excel (Redmond, WA, USA). 

Results

Five studies met the inclusion criteria and were included in this systematic review. These studies varied in design, patient populations, RIPC protocols, comparators and renal outcomes measured. The studies ranged from randomized controlled trials (RCT) to a propensity score-matched cohort study and included both robotic and laparoscopic partial nephrectomy patients. A summary of the characteristics of included studies is provided in Table 2. 

**Table 2:** Characteristics of included studies RIPC: remote ischemic preconditioning, RAPN: robot-assisted partial nephrectomy, RCT: randomised control trial, RCC: renal cell carcinoma, RALPN: robotically assisted laparoscopic partial nephrectomy, ITMB: intrathecal morphine block, EGFR: estimated glomerular filtration rate, NGAL: neutrophil gelatinase-associated lipocalin, BOLD-MRI: blood oxygen level dependent-magnetic resonance imaging, MRI: magnetic resonance imaging

Study	Design	Population	Sample size			Intervention	Comparator	Primary Outcome	Main Findings
			Intervention Group (RIPC or bundle)	Control Group	Total sample size				
Omae et al. (2023) [14]	Propensity score-matched cohort	Adults with solitary renal tumor undergoing RAPN	53	53 (matched from 482 total)	106	3 cycles of 5-min ischemia RIPC	No RIPC	Post-op eGFR	No significant difference; longer hospital stay in RIPC
Chae et al. (2022) [15]	Double-blind RCT	RCC patients undergoing RALPN	40 (RIPC + ITMB bundle)	40 (non bundle)	80	RIPC + intrathecal morphine block	No bundle	Lowest eGFR within 48 hrs	Significant renal protection, less pain, shorter stay
Heuzeroth et al. (2024) [16]	RCT	Partial nephrectomy patients	8	7	15	3 cycles 5-min ischemia RIPC	Sham procedure	Urinary NGAL, BOLD-MRI	Reduced NGAL, no sig. MRI changes
Chung et al. (2024) [17]	RCT	Partial nephrectomy patients	41	40	81	4 cycles 5-min ischemia RIPC	No RIPC	Serum creatinine	Reduced serum creatinine rise
Hou et al. (2017) [18]	RCT	Partial nephrectomy patients	40	20	60	3 cycles of 5-min upper-limb ischemia	No RIPC	Serum creatinine, eGFR, urinary NGAL	RIPC group showed significantly attenuated rise in serum creatinine, trend toward higher early eGFR, and reduced urinary NGAL. No RIPC-related adverse events reported.

Omae et al. [14] conducted a propensity score-matched cohort study involving 106 patients undergoing robot-assisted laparoscopic partial nephrectomy. The intervention group received three cycles of lower limb ischemia-reperfusion via blood pressure cuff inflation. Despite rigorous matching, no significant differences were observed in postoperative eGFR at multiple time points or perioperative outcomes. Interestingly, the RIPC group had a modestly longer hospital stay by approximately 1.3 days. Risk of bias was rated moderate due to the non-randomized design, potential residual confounding and missing data, limiting the strength of conclusions about RIPC efficacy. 

Chae et al. [15] conducted a high-quality double-blind RCT evaluating a multimodal intraoperative bundle of RIPC combined with intrathecal morphine block in 80 patients undergoing robot-assisted laparoscopic partial nephrectomy. The bundle group demonstrated significantly higher early postoperative eGFR values and reduced eGFR decline within 48 hours post-surgery compared to controls. Secondary outcomes favoured the bundle group, with decreased pain scores, lower opioid consumption, and shorter hospital stays. No serious adverse events occurred. The study’s rigorous design and low risk of bias strengthen the evidence supporting combined RIPC and analgesic strategies to enhance early renal recovery. 

Heuzeroth et al. [16] investigated RIPC in a small RCT of 15 patients using functional blood oxygenation level-dependent MRI and urinary NGAL as a biomarker of tubular injury. The RIPC group showed significantly reduced urinary NGAL levels over five postoperative days, indicating less tubular damage. Blood oxygen level dependent (BOLD)-MRI revealed smaller but non-significant differences in renal oxygenation changes compared to controls. The study was deemed at moderate risk of bias due to its small sample size and unclear blinding of imaging assessors, though it supports the feasibility of BOLD-MRI as a non-invasive modality to detect RIPC effects. 

Chung et al. [17] randomized 81 patients undergoing partial nephrectomy to RIPC or control and measured serum creatinine and eGFR postoperatively. The RIPC group had a significantly attenuated rise in serum creatinine, suggesting improved renal protection. While the incidence of acute kidney injury was lower in the RIPC group, this did not reach statistical significance. Secondary renal function measures favoured RIPC but without statistical significance. The study had a low to moderate risk of bias, with well-described randomization and blinding, though outcome assessor blinding was unclear. 

Hou et al. [18] conducted an RCT evaluating the effect of RIPC on renal function in patients undergoing partial nephrectomy under general anesthesia. The study included 60 adult patients, randomized to either RIPC (three cycles of five-minute ischemia applied to the upper limb) or control (no RIPC). Primary outcomes included early postoperative renal function assessed by serum creatinine, eGFR, and urinary NGAL. Hou et al. reported a significant attenuation in the postoperative rise of serum creatinine and a trend toward higher early eGFR in the RIPC group compared with controls (P < 0.05) [18]. Urinary NGAL levels were lower in the RIPC group, indicating reduced tubular injury. No adverse events related to RIPC were observed. Risk of bias was assessed as low, with adequate randomization, allocation concealment, and blinding of outcome assessors. 

Exploratory meta-analysis of eGFR was performed. Two randomized trials reported extractable eGFR data (Hou et al. [18] - eGFR at three months; Chae et al. [15] - lowest eGFR within 48 h). Pooled by a random-effects model, the mean difference in eGFR (intervention − control) was +7.7 mL/min/1.73 m² (95% CI 0.65 to 14.69; p = 0.032). Heterogeneity was moderate (Q = 2.41, df = 1, p = 0.12; I² = 58.4%, τ ≈ 4.0). Because pooled studies measured eGFR at different timepoints and used dissimilar RIPC strategies (RIPC alone vs RIPC + intrathecal morphine bundle), this pooled estimate is exploratory only. We therefore emphasise effect sizes and confidence intervals for individual studies in the narrative synthesis rather than treating the pooled point estimate as definitive. For serum creatinine, a quantitative pooling was not possible across all RCTs because either comparable timepoint data or SDs were not available in every trial. 

Across these heterogeneous studies, evidence for RIPC’s renoprotective effect during partial nephrectomy is mixed. High-quality RCTs with rigorous blinding such as Chae et al. [15] show beneficial effects on early renal function and postoperative recovery, while propensity-matched cohort studies and smaller RCTs report inconclusive or non-significant results. Risk of bias varies, with randomized trials generally at low to moderate risk, and non-randomized studies at higher risk due to confounding and missing data. Overall, while RIPC appears safe and feasible, the current evidence base is limited by small sample sizes, methodological heterogeneity, and inconsistent outcome measures, warranting further large-scale, standardized trials. Randomized controlled trials [15-18] generally demonstrated low to moderate risk of bias, with adequate randomization, allocation concealment, and blinding, though some lacked clarity on blinding of outcome assessors. Omae et al.’s [14] non-randomized study carried a moderate risk due to residual confounding and missing data. Collectively, these assessments indicate that methodological quality was generally acceptable, but with some limitations in study design and precision that may affect the interpretation of pooled estimates. A summary of the risk of bias assessment can be seen in Table 3.

**Table 3:** Summary of the risk of bias assessment ROBINS-I: Risk of Bias in Non-randomised Studies - of Interventions, RoB 2: Risk of Bias 2, RCT: randomized controlled trial, eGFR: estimated glomerular filtration rate, PS: propensity score, FU: follow-up

Study	Design	RoB Tool	Randomization / Confounding	Deviations from Intended Interventions	Missing Data	Outcome Measurement	Selective Reporting	Overall Risk
Chung et al., 2021 [17]	RCT	Cochrane RoB 2	Low risk (computer-generated, concealed)	Low risk (blinded)	Low risk	Low risk	Low risk	Low Risk
Omae et al., 2023 [14]	Observational, PS-matched cohort	ROBINS-I + Newcastle-Ottawa Scale	Moderate risk (retrospective design)	Low risk	Serious risk (missing eGFR in controls)	Low risk	Low risk	Moderate Risk
Heuzeroth et al., 2024 [16]	RCT (pilot, small sample)	Cochrane RoB 2	Low risk	Low risk	Low risk	Low risk	Low risk	Low Risk
Chae et al., 2022 [15]	RCT	Cochrane RoB 2	Low risk (balanced arms, good concealment)	Low risk	Low risk	Low risk	Low risk	Low Risk
Hou et al; 2017 [18]	RCT	Cochrane RoB 2	Some concerns (randomization computer-generated but allocation concealment unclear)	Low risk (blinded anesthesiologists, surgeons, data collectors; sham control used)	Low risk (5/65 lost to FU, small, balanced)	Low risk (objective lab/imaging outcomes, blinded analysis)	Some concerns (trial registered but unclear alignment with reported outcomes)	Some concerns

The certainty of evidence was evaluated using the Grading of Recommendations Assessment, Development and Evaluation (GRADE) framework [19] across five domains: risk of bias, inconsistency, indirectness, imprecision, and publication bias. Among the five included studies, Chae et al. [15] was rated as providing high-certainty evidence due to its robust randomized design, low risk of bias, and adequate sample size. Two randomized controlled trials, Chung et al. [17] and Heuzeroth et al [16], were downgraded due to imprecision from limited sample sizes and, in one case, reliance on surrogate imaging outcomes. One observational study, Omae et al. [14], was downgraded for serious imprecision and moderate risk of bias inherent to retrospective design. Overall, the certainty of evidence for the effect of RIPC on renal outcomes in partial nephrectomy ranged from low to high, with most studies falling within the low to moderate category. A summary of the GRADE assessment can be seen in Table 4. 

**Table 4:** Grading of Recommendations Assessment, Development and Evaluation (GRADE) summary RCT: randomized control trial, PS: propensity score, CI: confidence interval

Study	Study Type	Risk of Bias	Inconsistency	Indirectness	Imprecision	Publication Bias	Certainty of Evidence
Chung et al. [17]	RCT	Low	Not serious	Not serious	Serious (underpowered)	Unlikely	Moderate
Omae et al. [14]	Observational (PS-matched)	Moderate	Not serious	Not serious	Serious (wide CI, small n)	Possible	Low
Heuzeroth et al. [16]	RCT (pilot)	Low	Not serious	Some concerns (surrogate markers only)	Serious (very small n)	Unlikely	Low
Chae et al. [15]	RCT	Low	Not serious	Not serious	Not serious	Unlikely	High
Hou et al. [18]	RCT	Some concerns	Not serious (single trial)	Not serious	Serious (small n, wide CI, limited clinical outcomes)	Possible	Low

We downgraded for imprecision when 95% confidence intervals crossed a predefined clinical decision threshold (chosen a priori as ±5 mL/min/1.73 m² for eGFR). For example, the pooled 95% CI of +0.65 to +14.69 spans values of marginal clinical relevance (near zero) and larger beneficial effects; therefore imprecision was considered serious despite a pooled point estimate favouring RIPC. Additionally, several trials were small (pilot trials with n < 20), so the total information size was limited relative to the optimal information size.

Discussion

This systematic review evaluated the efficacy of RIPC in reducing renal injury following partial nephrectomy, focusing on urinary biomarkers and long-term kidney function outcomes. The five included studies collectively provide mixed evidence regarding the renoprotective effects of RIPC in this surgical context. 

Among the reviewed studies, high-quality randomized controlled trials such as Chae et al. [15] demonstrated promising results. This study’s use of a combined intraoperative bundle RIPC paired with intrathecal morphine yielded significant improvements in early postoperative renal function, reduced pain scores, decreased opioid consumption and shorter hospital stays. These findings suggest that RIPC may contribute to renal protection when integrated into a multimodal perioperative strategy. Conversely, smaller RCTs and observational cohort studies, including Omae et al. [14] and Heuzeroth et al. [16], reported either no significant effect or modest biomarker improvements without clear functional benefit, highlighting potential variability in RIPC efficacy. 

A recent systematic review and meta-analysis by Zhang et al. [20], which included 11 RCTs across renal surgery and transplantation, also reported only modest renoprotective effects of RIPC. Specifically, they observed a statistically significant but transient improvement in eGFR at three months after kidney transplantation, with minimal impact on serum creatinine and no sustained long-term benefits. These findings are consistent with our review, which similarly identified short-term improvements in renal biomarkers and function following partial nephrectomy but no durable renal protection. Importantly, Zhang et al.’s review synthesized heterogeneous surgical populations, whereas our analysis focuses specifically on partial nephrectomy, providing a more targeted perspective [20]. 

The variable efficacy of RIPC observed in our study highlights that its renoprotective effects in partial nephrectomy remain uncertain. While early enthusiasm for RIPC stemmed from cardiothoracic trials, subsequent large multicentre studies failed to reproduce clinical benefits, underscoring the importance of patient population, anaesthetic technique, and procedural context. In renal surgery, prior RCTs have reported similarly mixed findings some demonstrated reductions in biomarkers of acute kidney injury, while others found no significant differences in postoperative renal function. Our results are consistent with this heterogeneity, as we observed transient reductions in NGAL and CysC but no sustained improvement in GFR at follow-up. This suggests that the potential renoprotective effect of RIPC may be subtle and influenced by perioperative variables such as ischemia duration, baseline renal reserve, and anaesthetic regimen. Future studies in partial nephrectomy should therefore adopt standardized RIPC protocols and incorporate clinically meaningful renal endpoints to clarify whether the intervention can translate into durable benefit. 

Limitations and Strengths of Included Studies 

A major limitation across the included studies is the substantial heterogeneity in study design, RIPC protocols, surgical techniques, and outcome reporting, which significantly impairs the ability to draw definitive conclusions. Variability was observed in several key aspects: the number and duration of ischemic cycles, the limb used for preconditioning, timing of RIPC administration relative to surgery and the use of adjunct interventions such as intrathecal analgesia. Such inconsistencies create performance and detection biases and challenge any attempt to isolate the effect of RIPC as an independent variable. Moreover, the inclusion of both robotic and laparoscopic surgical approaches introduces further variation in ischemia times, surgical manipulation, and baseline stress response, all of which may modulate susceptibility to ischemia-reperfusion injury and influence renal outcomes. Outcome measures also varied, with some studies focusing on urinary biomarkers, others on early postoperative eGFR, and few assessing long-term renal function or incidence of chronic kidney disease. The timing of outcome assessment also ranged from 24 hours to several months postoperatively, complicating cross-study comparisons. This lack of standardization hinders meta-analytic synthesis and undermines external validity. Moving forward, the adoption of uniform RIPC protocols such as detailing cycle number, duration, limb selection and timing, as well as standardized outcome definitions and measurement intervals, will be critical to facilitate reproducibility, comparability, and ultimately, clinical translation of findings in this field. 

A limitation of this review is that grey literature sources and clinical trial registries were not systematically searched. Consequently, relevant unpublished or ongoing studies may have been missed, introducing the potential for publication bias. Nevertheless, this review benefits from a rigorous search strategy, comprehensive inclusion of recent literature, and critical appraisal of study quality using validated tools. The synthesis highlights key gaps and informs future research directions. 

Potential Mechanisms and Clinical Implications 

RIPC is hypothesized to confer protection through systemic release of humoral factors and activation of neural pathways that mitigate ischemia-reperfusion injury, including modulation of inflammatory responses, reduction of oxidative stress, and enhancement of cellular survival pathways [21]. The reduced urinary NGAL levels reported by Heuzeroth et al. [16] support a biological basis for tubular protection, though functional imaging outcomes were less definitive. Notably, combining RIPC with analgesic techniques, as in Chae et al.’s study [15], may synergistically reduce perioperative stress and inflammation, potentially amplifying renoprotective effects. 

Clinically, these findings suggest that RIPC is a safe, low-cost, non-invasive intervention with potential benefits, particularly when applied as part of a broader protective strategy. However, the inconsistent evidence underscores the need for standardized RIPC protocols, optimal timing and dosing, and well-defined clinical endpoints. Given its non-invasive nature and low cost, RIPC holds appeal as a scalable intervention. Nevertheless, without standardized implementation or proven long-term benefit, its routine clinical use cannot yet be justified 

Recommendations for Future Research 

To clarify RIPC’s role in protecting renal function after partial nephrectomy, future large-scale randomized trials should employ standardized and reproducible RIPC protocols, detailing ischemic cycle timing, duration and limb selection. Studies should also incorporate multimodal strategies that may potentiate renoprotection. Sensitive urinary biomarkers such as NGAL and robust long-term functional outcomes such as eGFR trajectories should be measured. Loss to follow-up to reduce bias can be minimised by ensuring adequate blinding and allocation concealment. Furthermore, patient subgroups that might derive the most benefit, such as those with pre-existing renal impairment or complex tumors requiring longer ischemia should be explored. 

## Conclusions

Remote ischemic preconditioning is a safe, low-cost, and technically simple intervention that shows potential for reducing short-term renal injury in patients undergoing partial nephrectomy. However, the evidence remains inconclusive, with benefits largely limited to early postoperative improvements in eGFR or urinary biomarkers, and no consistent demonstration of sustained long-term protection. These findings echo broader surgical literature, where enthusiasm for RIPC has been tempered by mixed trial results and protocol variability. At present, routine clinical implementation cannot be recommended. Future adequately powered randomized trials using standardized RIPC protocols, standardized outcome measures, and extended follow-up are essential to determine whether RIPC can meaningfully improve renal outcomes, particularly in high-risk subgroups such as patients with baseline renal impairment or prolonged ischemia times.

## Appendices

Appendix A: Search strategy for various databases

**Table 5:** Search strategy for the databases

Data Base	Search Strategy	No. of articles
PUBMED	("partial nephrectomy" OR "nephron sparing surgery") AND ("remote ischemic preconditioning" OR "remote ischemic conditioning" OR RIPC) AND ("renal injury" OR "acute kidney injury" OR "AKI" OR "urinary biomarkers" OR "NGAL" OR "KIM-1" OR "eGFR" OR "estimated glomerular filtration rate" OR "serum creatinine" OR "kidney function") RIPC and Partial Nephrectomy	9
COCHRANE	("partial nephrectomy" OR "nephron sparing surgery") AND ("remote ischemic preconditioning" OR "remote ischemic conditioning" OR RIPC) AND ("renal injury" OR "acute kidney injury" OR AKI OR "urinary biomarkers" OR NGAL OR "KIM-1" OR "kidney function" OR "eGFR" OR "serum creatinine")	13
EMBASE	1. 'partial nephrectomy'/exp OR "partial nephrectomy".mp. OR nephrectomy.mp. 2. 'remote ischemic preconditioning'/exp OR "remote ischemic preconditioning".mp. OR RIPC.mp. 3. 'renal injury'/exp OR "renal injury".mp. OR "acute kidney injury".mp. OR AKI.mp. OR "kidney injury".mp. OR "renal function".mp. OR "kidney function".mp. 4. 'urine biomarker'/exp OR "urinary biomarker*".mp. OR "urine marker*".mp. OR NGAL.mp. OR "neutrophil gelatinase associated lipocalin".mp. OR KIM-1.mp. OR "kidney injury molecule 1".mp. OR IL-18.mp. OR NAG.mp. OR "N-acetyl-β-D-glucosaminidase".mp. OR "cystatin C".mp. OR "netrin-1".mp. OR MCP-1.mp. OR "monocyte chemoattractant protein-1".mp. 5. 1 AND 2 AND (3 OR 4) 6. limit 5 to human 7. limit 6 to english language 8. limit 7 to yr="2010 -Current" 9. limit 8 to (randomized controlled trial OR cohort study OR case control study)	56
SCOPUS	(TITLE-ABS-KEY("partial nephrectomy" OR "nephron sparing surgery")) AND (TITLE-ABS-KEY("remote ischemic preconditioning" OR "remote ischemic conditioning" OR RIPC)) AND (TITLE-ABS-KEY("renal injury" OR "acute kidney injury" OR AKI OR "urinary biomarkers" OR NGAL OR "KIM-1" OR "kidney function" OR "eGFR" OR "serum creatinine")	11
